# Bilateral MR imaging-guided high intensity focused ultrasound for the treatment of tremor-dominant Parkinson’s disease: first experience with 9 months follow up

**DOI:** 10.1186/2050-5736-3-S1-O4

**Published:** 2015-06-30

**Authors:** Ronald Bauer, Beat Werner, Stefan Hägele-Link, Georg Kägi, Florian Brugger, Nikolas Arne Wegener, Ernst Martin

**Affiliations:** 1 The Kantonsspital St. Gallen, St. Gallen, Switzerland; 2 Center MR Research, Zurich, Switzerland; 3 MR Research Center, Zurich, Switzerland

## Background/introduction

MR imaging-guided high intensity focused ultrasound (MRIgFUS) is a novel, noninvasive technique for the treatment of functional brain disorders through the intact human skull at millimeter precision. The ExAblate 4000 transcranial MRIgFUS system (InSightec, Haifa, Israel) uses a 1024-element phased array transducer, which is attached to the patient’s head via a standard stereotactic frame situated inside a 3T MRI scanner, and is CE certified for interventions in the thalamus, subthalamus and pallidum.

## Methods

Case Report: We report the case of a 45 years old male patient with medication-resistant tremor dominant Parkinson’s disease (PD). Deep brain stimulation (DBS) was contraindicated due to a bipolar disorder.

Intervention: During MRIgFUS treatment the target was visually focused by MR-image guidance. In a first step, the correct location of sonication was verified with low, non-ablative energy, and targeted in the pallido-thalamic tract (fasciculus thalamicus) of the subthalamic area.

Repeated sonications each lasting 15 to 25 seconds were delivered with stepwise increased acoustic energy up to 13200 J to create thermocoagulations under real-time MR-thermometry. The sonications resulted in peak temperature of 60°C at the target points producing a thermal lesion of approximately 3x3x4mm in size. Circulating de-gassed water between the helmet shaped transducer and the patient’s head provided acoustic coupling and head cooling.

After each sonication the patient was interviewed and neurologically tested. The size of the lesion was closely monitored by MR-imaging during and directly after treatment, and again after 48 hours and at one month.

## Results and conclusions

Clinically, MRIgFUS intervention resulted in a prompt and complete suppression of the tremor, improvement of gait, posturing and rigor (9 months follow up) leading to significant improvement of quality of life. The motor part III of the Unified Parkinson’s Disease Rating Scale (UPDRS) after the intervention decreased from 56 to 14 out 108 possible points (higher values meaning more impairment). The patient showed a fortiforcation of bipolar disorder as a transient side effect in relation to dynamic of surrounding oedema.

MRIgFUS is a novel, non-invasive technique for the treatment of Parkinson’s disease that does not use ionizing or radioactive radiation. The technique has been proven successful worldwide in the treatment of functional brain disorders, such as neuropathic pain, movement disorders and neuropsychiatric diseases in over 180 patients.

Our first experience in treating 4 Parkinson patients and 2 patients with essential tremor suggests that MRIgFUS will become a safe and effective treatment method for patients with movement disorders.

Written informed consent was obtained from the patient for publication of this abstract and any accompanying images

**Figure 1:**
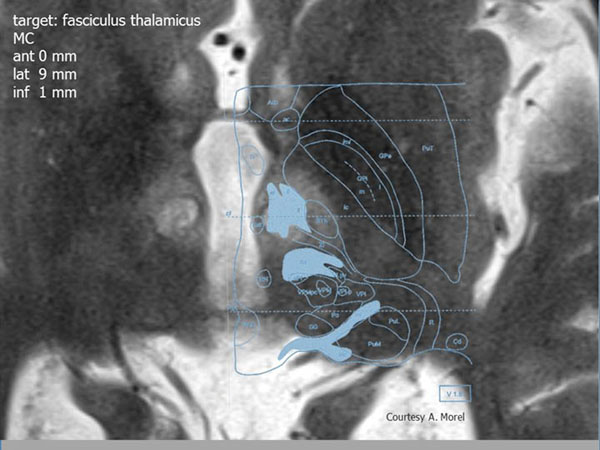
Bilateral pallido-thalamo-tractotomy in Parkinon’s Disease 48 h post intervention

